# Three-dimensional analysis of modeled facial aging and sexual dimorphism from juvenile to elderly age

**DOI:** 10.1038/s41598-022-26376-8

**Published:** 2022-12-17

**Authors:** Jana Velemínská, Lenka Kožejová Jaklová, Karolina Kočandrlová, Eva Hoffmannová, Jana Koudelová, Barbora Suchá, Ján Dupej

**Affiliations:** Department of Anthropology and Human Genetics, Faculty of Science, Charles University, Viničná 7, 128 43 Prague, Czech Republic

**Keywords:** Musculoskeletal models, Computational models, Ageing

## Abstract

A detailed understanding of craniofacial ontogenetic development is important in a variety of scientific disciplines dealing with facial reconstruction, forensic identification, ageing prediction, and monitoring of pathological growth, including the effect of therapy. The main goals of this study were (1) the construction of the facial aging model using local polynomial regression fitting separately for both sexes, (2) evaluation of the aging effect not only on facial form as a whole but also on dimensions important for clinical practice, and (3) monitoring of the development of shape facial sexual dimorphism. Our study was based on the form and shape analysis of three-dimensional facial surface models of 456 individuals aged 14–83 years. The facial models were obtained using a structured light-based optical scanner and divided (for some analyses) into four age categories (juveniles, young adults, middle adults, and elderly adults). The methodology was based on geometric and classic morphometrics including multivariate statistics. Aging in both sexes shared common traits such as more pronounced facial roundness reducing facial convexity, sagging soft tissue, smaller visible areas of the eyes, greater nose, and thinner lips. In contrast to female faces, male faces increase in size until almost 30 years of age. After the age of 70, male facial size not only stagnates, like in females, but actually decreases slightly. Sexual dimorphic traits tended to diminish in the frontal and orbitonasal areas and increase in the gonial area.

## Introduction

Accurate and complex evaluation of facial morphology is dependent on the understanding of ontogenetic facial development, including variability, sexual dimorphism, facial expression and pathological deformity. The knowledge of the age- and sex-related qualitative and quantitative characteristics provides useful information both in medicine and forensic sciences; recently it has been useful in connection with the perception of attractiveness, in ethology and the morphological divergence of the human face among populations^1,2^.

Medical reference facial data are necessary for maxillofacial surgery, plastic surgery, genetics and orthodontists. With regard to pre- and post-operative treatment, the comparisons of facial differences between patients with craniofacial anomalies or syndromes with those fulfilling normative values, as well as comparisons between different age and sex groups, are important in deciding on an appropriate therapeutic course^3,4^. The data are also of great importance in traumatology, including facial reconstruction by transplantation in different periods of an individual’s life^5^. Craniofacial morphology also plays an important role in the accurate diagnosis of various rare genetic disorders that are associated with facial dysmorphology. Alterations of facial morphology commonly represent the initial diagnostic sign, which triggers subsequent interdisciplinary diagnostic examinations and may thus lead to patient diagnosis^6^. From a medical point of view, it is important to know not only the developmental changes of the face as a whole, but also the specific changes of precisely defined facial dimensions for routine clinical practice. This is the reason why we evaluated the development of the face from juvenile to late adulthood using both geometric morphometric and classical morphometric methods.

Forensic facial identification is a very difficult task, which requires less subjective and more empirical approaches^7^. Successful classification and recognition according to facial features are essential in many forensic applications, e.g., facial reconstruction, forensic facial identification, aging prediction, or facial recognition. Knowledge of senescence-related facial changes with regard to sexual dimorphism is particularly valuable especially for the identification of long-term missing persons and facial reconstruction^8^. Facial ageing in the forensic context is necessary both for the dead and the living. For the dead, ageing estimations principally model the biological profile, which can be compared to missing persons. For living children and juveniles, ageing approaches help to solve judicial or civil problems concerning the age of minors in regard to questions of adoption, imputability and pedopornography. For adults, these problems contribute to age estimation in association with pensionable age determination, invalid identification documents and other similar matters^7^.

Facial age-progression is a continuous and dynamic process that does not occur at a uniform pace. Aging trajectories are not constant throughout a human life, and differences are apparent both between different age-stages (i.e. juvenile vs. adult) and sexes^9,10^. During the growth period, the formation of facial appearance mostly concerns the development of facial form (shape and size); there is an codependent relationship between bone morphology and the volume of the soft-tissue envelope^11^. By contrast, later stages of adulthood are characterised by a combination of changes in the soft tissue (such as changes in the state of elastin and collagen fibers), with bone loss in specific areas of the facial skeleton^12,13^. That facial soft tissue and underlying bone structure may change dynamically over a life span thus suggests that the assumptions based on visible facial sexual dimorphism in a younger sample may not be usefully generalized to older adult samples^14^.

Sexual dimorphism in facial characteristics greatly contributes to the variability of the human face and exists from prenatal development^10^. Although in some cases sex could already be estimated from the infant face, the degree of sexual dimorphism changes as a function of age^10,15^. The growth rate is not constant throughout ontogenetic development and differences are also apparent between the sexes, especially during adolescence, when sex hormones play a major role in facial appearance^16^. The generally accepted view is that sexual dimorphic facial traits become more apparent after 13 years of age and result from the different growth trajectories in males and females^9^.

Many previous studies concerning the growth and development of facial morphology, as well as facial sexual dimorphism during aging, were based on the evaluation of two-dimensional (2D) data, i.e. photographs or X-ray images^10,17–21^. These studies have some specific limitations—e.g. they are focused only on particular regions of the face (mouth, nose, eyes), and evaluate linear distances, ratios, angles, areas and volumes. Although this information can be useful in some of the above-mentioned fields, they can not describe the face as a complex structure (in terms of the relationship between each of the facial parts). Recently, 3D imaging systems have become widespread and are commonly used instruments in the evaluation of facial soft tissue because of their objective and accurate analysis of the whole facial surface^22^. Nevertheless, the studies are concentrated mainly on childhood, juvenile and adolescent periods^23–28^ and young adults^29–31^. The studies that focused on the development of facial sexual dimorphism in later stages of human life are rare^8,32^.

Our study provides an assessment of the development of the face as a whole with regard to sex and based on the developmental trajectories for the age ranges between 14 and 83 years. This methodology is complemented by scatterplot data, which show the development of centroid size during aging, and colour maps that illustrate both the specific age-related differences in the individual areas of the facial form, as well as the development of facial shape sexual dimorphism. The benefit of the study is its continuity with the study of age-related differences in the sexual dimorphism of cranial shape^33^. The studied material also used a recent Czech population, with the same age categories (with the exception of the juvenile category) and a methodology based on the superimposition of the average skull shapes using colour-coded maps. This study also includes tables showing the development of facial dimensions intended primarily for clinical practice.

## Material

The 3D virtual facial models were obtained from 2009 to 2019 from 456 individuals with Czech nationality; they did not have congenital anomalies or craniofacial trauma. A total of 250 women and 206 men aged 14–83 years were divided into 4 age categories as described in Table 1: juveniles to 19 years (0), young adults from 20 to 40 years (I), middle adults from 41 to 60 years (II), and elderly adults from 61 to 83 years (III). Adult subjects and legal guardians signed informed written consent forms.

**Table 1:** Age categories used in the study.

Category	Age (yrs)	Females (n)	Mean age (yrs) + SD	Males (n)	Mean age (yrs) + SD
Juveniles (0)	14–19	50	17 ± 1.34	54	16 ± 1.33
Young adults (I)	20–40	69	27 ± 5.45	63	27 ± 5.39
Middle adults (II)	41–60	80	50 ± 4.78	64	51 ± 4.96
Elderly adults (III)	61–83	51	70 ± 6.55	25	68 ± 5.18

## Methods

The Vectra 3D high-resolution optical scanner (Canfield Scientific, Inc., USA) was used to capture 3D facial models. All individuals were seated, asked for a neutral facial expression, and captured from a frontal position. Exported models were edited using Rapidform 2006 software (Inus Technology, Inc., South Korea). The editing consisted of trimming away those parts of the image that contained the hair, ears, and neck, and correcting small holes and vertex errors. As a last step, each model was decimated to approximately 25 k vertices. The prepared models were imported into Morphome3cs II software (http://www.morphome3cs.com/) for further processing and morphometric analysis. Such models generally lack semantic consistency across vertices. Before geometric morphometry can be applied, the models must be resampled in a homologous way, turning the vertices into quasi-landmarks. The correspondending Coherent Point Drift-Dense Correspondance Analysis (CPD-DCA) search algorithm was used to obtain vertex homology^28^.

The first step consisted of the application of 9 landmarks (Table 2, landmarks 1–9) located on the significant anatomical structures on the 3D facial models in a specific order. A random surface model was selected as a template (base mesh) to define and set its topology for all the models. The meshes were resampled using CPD-DCA, which also discarded areas that were not present in all the meshes. Generalized Procrustes Analysis (GPA) was performed on the resampled meshes to suppress any residual pose inconsistencies. Size was restored after GPA to yield form. As the last step, Principal Component Analysis (PCA) provided a representation of the data in a reduced dimension for further statistical analysis. In addition, we also compared facial sexual dimorphism after size normalization in all four age categories using the same methods.

**Table 2:** Definition of the landmarks used in the study.

LM	Definition
Endocanthion (2—dx, 3—sin)	The inner point of the eye fissure at the junction of the eyelids
Exocanthion (1—dx, 4—sin)	The outer point of the eye fissure at the junction of the eyelids
Palpebrale superius (12—dx, 14—sin)	Point in the middle of the upper line of the eye
Palpebrale inferius (13—dx, 15—sin)	Point in the middle of the lower line of the eye
Nasion (5)	Point in the medial plane of the deepest concavity on the nasolabial suture
Pronasale (6)	Most anterior midline point on the nose tip
Alare (10—dx, 11—sin)	The most lateral point on each alar contour
Subnasale (16)	The most inferior point of the nose in the medial plane between columella and upper lip
Chelion (7—dx, 8—sin)	The point on the outer edge of the mouth where the lower vermilions meet at the outer corner of the mouth
Labiale superius (17)	Midpoint of upper vermilion line
Labiale inferius (18)	Midpoint of lower vermilion line
Pogonion (9)	The most protruding midpoint on the edge of the protuberantia menti on the anterior surface of the chin
Zygion (19—dx, 20—sin)	The most laterally located part of the zygomatic arch

The aging model was constructed using local polynomial regression fitting^34^ in the space of the first 200 principal components for each sex separately. Because the model was fitted to principal component scores, a minor loss of information could be expected. The used principal components accounted for over 99.7% of the total variability, so that loss is deemed negligible. Facial models for specific ages were synthesized by adding up the predicted 200 PC contributions to the mean form.

For each model, we also determined its centroid size (CS) and plotted it as a function of age. An aging trajectory was constructed using local polynomial regression fitting along with a 95% confidence region. This model was constructed for each sex separately.

Colour-coded distance maps were used to visualize and quantify facial form differences between average age categories 0 and I, I and II, and II and III. Generally, the more protrusive (locally inward) parts of the shell are coded in red, the more deeply (locally outward) situated parts are coded in blue, and the parts with no differences are marked in green. Depending on the resulting *p*-value, the facial areas were coloured into shades of blue and grey areas (significant differences were coded in shades of blue, depending on the *p*-value)^28^.

To visualise and quantify sex shape differences, the male and female average faces were constructed for each age. The distances of homologous vertices were computed and projected on the local surface normal to filter out any tangential shifts. These distances were displayed on the surface by colour-coding^35^. Red denotes that a particular area of the male face was located in front of the female face after superimposition, while blue indicates the converse condition. For statistical visualisation, we calculated the same normal-projected vertex distances from a common surface. Two-sample t-tests were performed on these distances in each vertex by sex, presenting the *p*-values as colour codes on the facial model.

**Figure 1:**
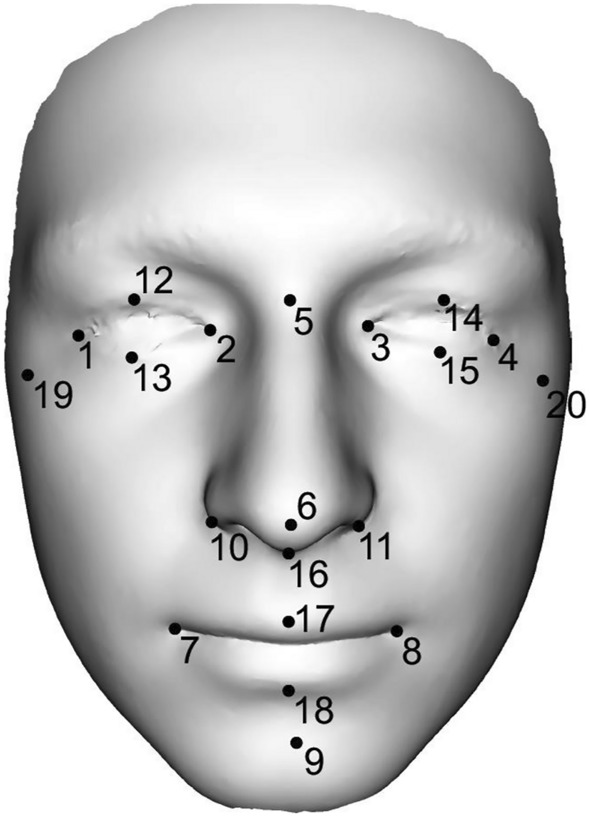
Representation of 20 landmarks on the 3D facial scan. The following dimensions were measured between the individual landmarks: ocular width (1–2, 3–4), ocular height (12–13,14–15), intercanthal width (2–3), biocular width (1–4), distances between exocanthion–nasion (1–5, 4–5), nasal length (5–6), nasal width (10–11), nasal depth (6–16), philtrum height (16–17), nasal height (5–16), dimension between pronasale–pogonion (6–9), mouth width (7–8), mouth height (17–18), facial height (5–9), lower face height (9–16) and facial width (19–20).

The initial step for the classic morphometric measurement of the face consisted in the localization of 20 landmarks on the 3D facial models in the specific order (Fig. 1 and Tab. 2). Among the landmarks, 22 linear dimensions describing the individual facial parts were measured in each age group. To detect changes in facial morphology during aging and the differences in sexual dimorphism in our set of individuals, a two-way ANOVA group was conducted for each of the dimensions by sex and age. Tukey post-hoc tests were also performed. This analysis was conducted in PAST. The level of statistical significance was set at α = 0.05.

### Ethical approval

All methods were carried out in accordance with relevant guidelines and regulations. All experimental protocols were approved by IRB Charles University, Faculty of Science, approval number 2022/16.

### Informed consent

 Informed consent was obtained from all subjects and their legal guardian(s).

## Results

The results are divided into three parts, with the first dealing with the complex geometry of the face. Detailed morphological age-related facial changes were evaluated using aging trajectories in the space of centroid size and extracted synthesized facial models (for 15, 20, 30, 40, 50, 60, 70 and 80 years of ages) and using a superimposition method of average male and female faces and colour-coded maps. The second part is devoted to the development of sexual dimorphism of the facial shape; the third part to classic morphometry. We evaluated how facial dimensions change from the youngest to the oldest age categories. The statistical significance of sexual dimorphic difference was also analyzed.

### Modeled facial development from juvenile to elderly age

**Figure 2:**
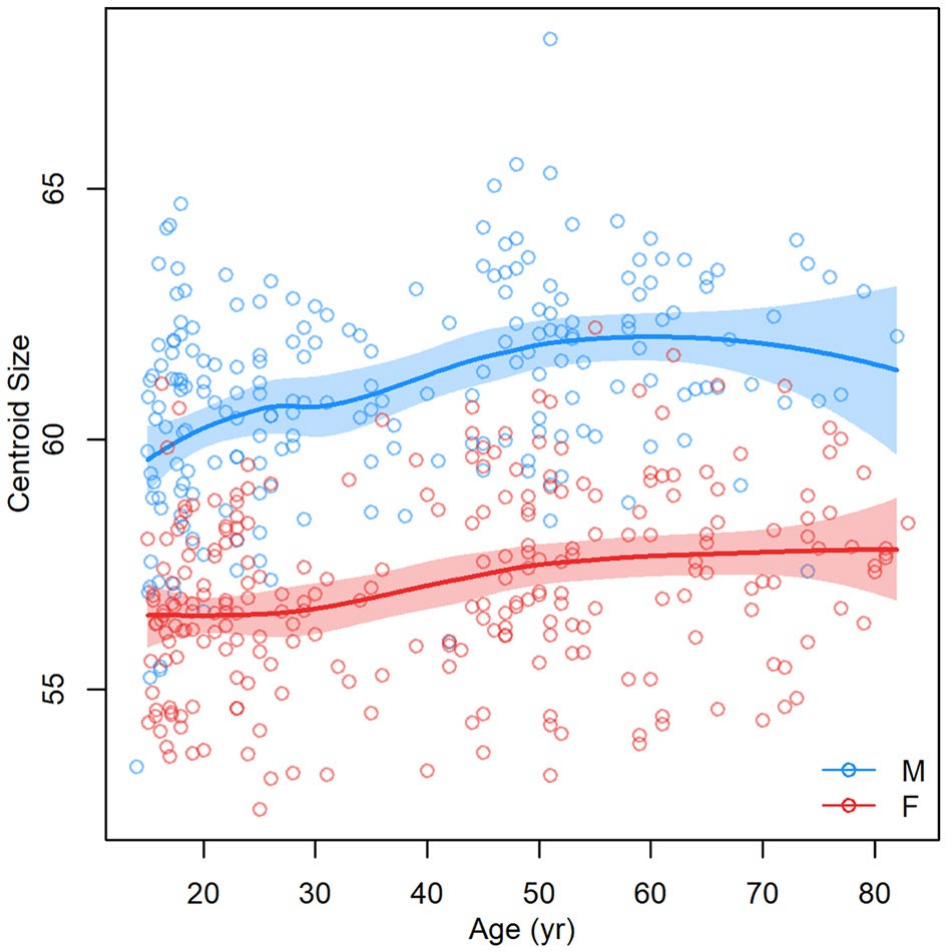
Dependence of facial centroid size on increasing age (the function was constructed separately for females and males as curves with a 95% confidence region).

**Figure 3:**
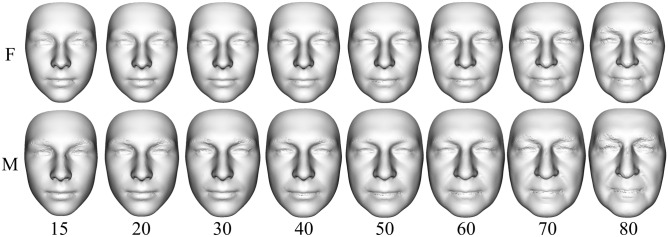
Predicted synthesized facial models for 15, 20, 30, 40, 50, 60, 70 and 80 years of ages created using 200 PC contributions to the mean form (F—females, M—males).

**Figure 4:**
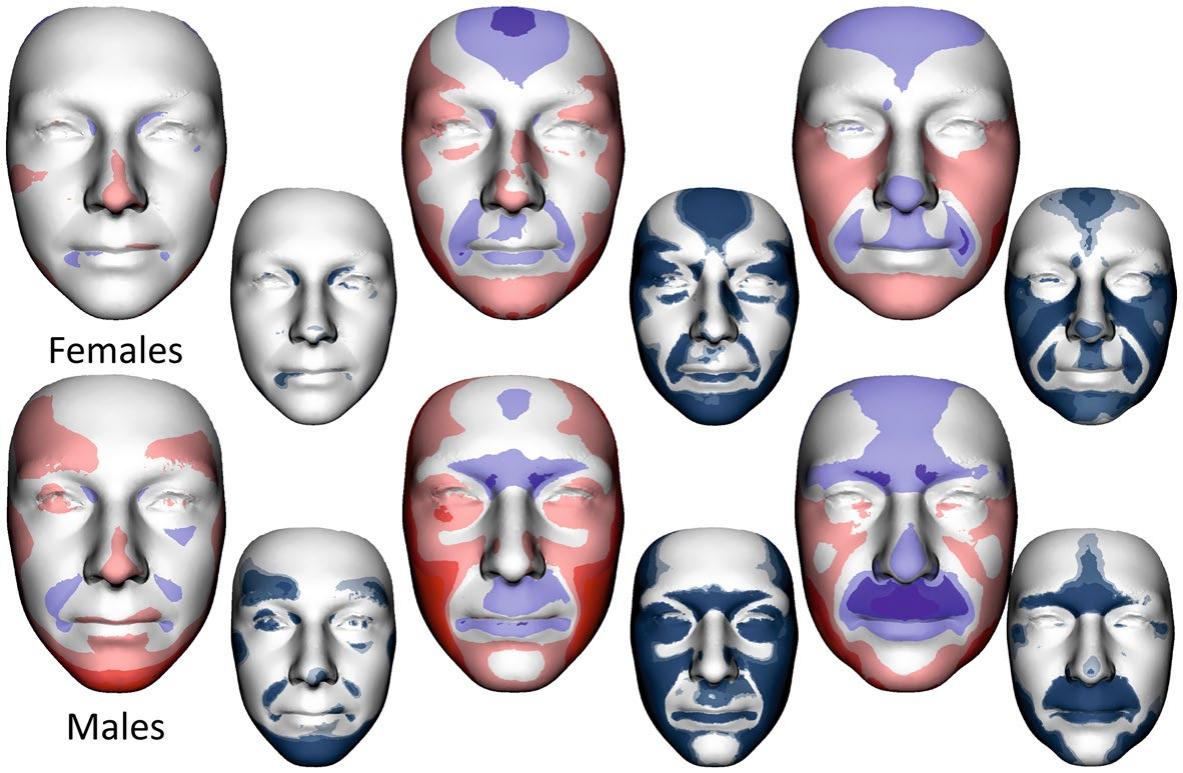
Colour-coded maps and shell distance significance maps describing average facial form differences between 0 and I, I and II, II and III age categories in females (upper row) and males (lower row). The most protrusive parts of the average faces are represented in red, whereas those that are situated deeper are coloured blue. The statistical significance of form differences was analysed per vertex and coded in shades of blue (significant differences) or grey (no significant differences) on the superimposed average faces.

Modeled facial development from juvenile to elderly age was visualized using the Figs. 2 and 3. In general, it can be stated that with increasing age, the faces of both sexes became larger and wider until the age of 70 (Figs. 3 and 4). A woman’s face changes very little by the age of 30, while a man’s face increases the most at that age. From 30 to 60 years of age, the faces of both sexes increase in size and widen slightly. Unlike women’s faces, men’s faces after the age of 60 shrink slightly. Aging after the age of thirty is manifested not only by the widening of the face, but also by the sagging of soft tissue and increased visibility of skin folds (Fig. 4).

Detailed morphological differences between age categories in females and males are described in Fig. 4. Colour-coded maps quantify facial form differences between juvenile age and young adulthood (0 and I), young and middle adulthood (I and II), and middle and elderly adulthood (II and III). The more protrusive (locally inward) parts in the older age category are coded in red; the more deeply (locally outward) situated parts in the older age category are in blue.

When we compared juvenile age and younger adulthood in women, there were no significant morphological changes, in contrast to the men. The male face stretched in the area of the lower jaw, highlighting the prominence of the superciliary arches and nasaltip. Between younger and middle adulthood, the aging trend was more similar for both sexes: widening of the face, reduction of the convexity of the forehead and both lips and highlighting of the sacs under the eyes. Retrusion of the forehead and area of both lips continued during elderly age, including the reduction of the prominence of the nasal tip. In addition, the protrusion of the superciliary arches and the glabella region decreased in males. Conversely, in females, facial widening continued, while in males, facial widening was apparent only in the area of the lower jaw.

### Development of facial sexual dimorphism during aging

**Figure 5:**
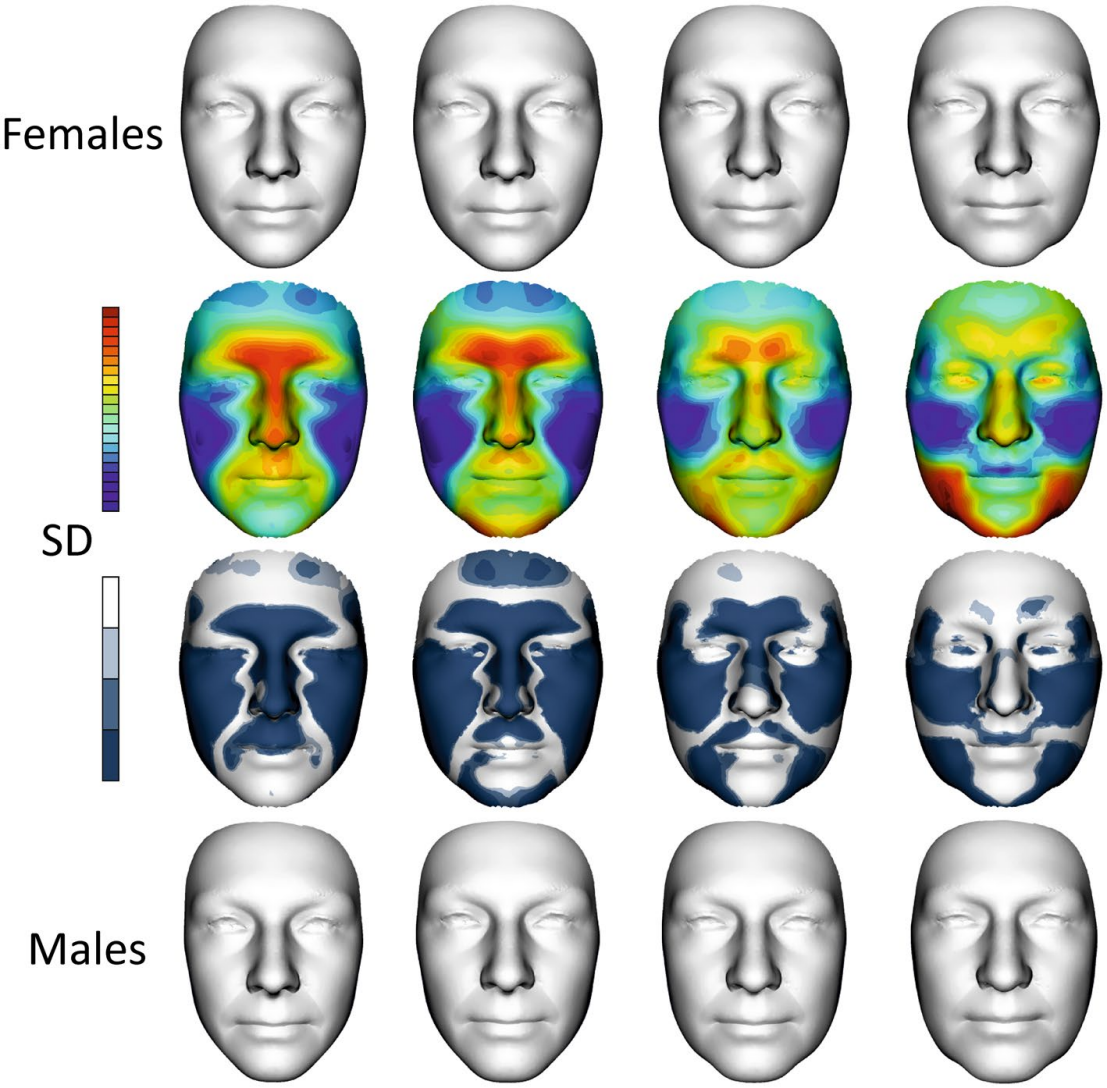
Development of sexual dimorphism of facial shape. The first row shows female average faces in all age categories, i.e., 0, I, II and III. The second row is a superimposition of the mean facial shapes of males and females in all age categories. The colour maps show the relative reciprocal locally inward/outward positions of the mean male and female facial shapes. Red denotes that a particular area of the male face was located in front of the female face after superimposition, while blue indicates the converse condition. The third row shows shell distance significance of sexual dimorphic maps in each age category. The last row shows average male faces in all age categories. (SD—shell distance).

Superimposition was used to evaluate shape differences between the average male face and the average female face from 14 to 83 years (Fig. 5) after size normalization. Visual comparisons are shown as colour-coded maps where the most protrusive or relatively greater parts of the average faces are represented in red, while the parts which are situated deeper or are relatively smaller are coloured blue. The statistical significance of shape difference was coded in shades of blue (significant differences) or grey (no significant differences) on the superimposed average faces (in the lower row of smaller faces).

Generally, the average male models tended to have more protruded lower parts of the forehead, eyebrow ridges, nose, upper lip and area of the philtrum compared to the female average; however, these differences were substantially reduced as they approached category III. Conversely, the males had deeper eye regions in the first age category, which were simultaneously reduced to the deeper female eye position in the last age category.

When we dealt with the juvenile age category, the sexual dimorphism of the upper part of the frontal bone and primarily area of the mandible was less apparent than in young adulthood. Sexual dimorphism in young adulthood was the largest and significant in all the monitored areas of the face. In the first and second age categories, the nasal length, eyebrow ridges, and upper lip were more protruded in males, while the superior part of the forehead and the cheeks were more protruded in females. Protruded cheeks and a more rounded face in females seemed to be the most stable sexual dimorphic features during aging (see maps of significance of all investigated categories).

Male protrusion of the chin was typical and largest in the second age category from 20 to 40 years. Chin prominence decreased with aging, but the width of the male mandible region increased. This widening of the lower third of the male face was related to the loss of the jawline, which changed from fluent to more fragmented, especially in the last age category.

When we look at the significance maps of facial shapes from juvenile to elderly age (Fig. 5, 3rd row of faces), we can see that sexual dimorphism was reduced with increasing age in the forehead, nose, upper lip, and chin; conversely, it was the most stable in the cheek area. Sexual dimorphism of the mandible region started to develop after 20 years of age in the chin area. The lateral parts of the lower face had the most sexual dimorphic difference after 60 years of age, when chin prominence is diminished. When we evaluated the face as a whole, sexual dimorphism was the smallest in the last age category.

### Differences in facial dimensions between each age category and sex

Eneti The two-way ANOVA followed by Tukey’s post hoc test demonstrated a significant effect of aging (0, I, II, III) and sex (F, M) on the evaluated facial dimensions (Table 3). For more accuracy, the effect size was calculated. The following reference values were used for the partial Eta Squared effect size: small effect = 0.01; medium effect = 0.06; and large effect = 0.14. The results of Tukey’s post hoc test can be seen in Supplementary Table S1 in the material.

**Table 3:** Mean values and standard deviations of dimensions in individual age categories, including testing of differences between age categories with respect to sexual dimorphism (Two-way ANOVA), including effect size.

Dimensions	Dim. (mm)	T0	T1	T2	T3	T0	T1	T2	T3	Sex	Age group	Interaction
		Female				Male				*p*-value & effect size		
Ocular width (dx)	1–2	28.56 ± 1.70	27.89 ± 1.88	26.89 ± 2.01	24.82 ± 2.14	29.84 ± 2.20	29.14 ± 2.23	27.84 ± 2.18	25.66 ± 1.95	≤ 0.001***; 0.106	≤ 0.001***; 0.286	0.231; 0.002
Ocular width (sin)	3–4	28.47 ± 1.68	28.19 ± 2.24	27.14 ± 1.99	24.99 ± 2.27	29.55 ± 2.21	29.46 ± 2.06	28.01 ± 2.53	25.81 ± 1.59	≤ 0.001***; 0.088	≤ 0.001***; 0.254	0.300; 0.002
Intercanthal width	2–3	32.94 ± 2.68	32.33 ± 2.84	33.73 ± 3.21	37.03 ± 3.76	34.09 ± 2.40	34.13 ± 2.64	36.21 ± 4.23	37.02 ± 3.97	≤ 0.001***; 0.036	≤ 0.001***; 0.163	0.033*; 0.016
Biocular width	1–4	88.70 ± 3.74	87.26 ± 3.64	86.97 ± 3.82	86.26 ± 3.19	92.04 ± 4.02	91.47 ± 3.82	91.41 ± 3.89	88.15 ± 3.19	≤ 0.001***; 0.218	≤ 0.001***; 0.053	0.039*; 0.014
Ocular height (dx)	12–13	11.08 ± 1.67	9.90 ± 1.34	9.55 ± 1.45	9.06 ± 1.43	10.42 ± 1.70	9.76 ± 1.53	9.62 ± 1.38	9.30 ± 1.41	0.909; 0.000	≤ 0.001***; 0.116	0.135; 0.011
Ocular height (sin)	14–15	11.00 ± 1.74	9.93 ± 1.48	9.39 ± 1.41	8.64 ± 1.33	10.42 ± 1.30	9.69 ± 1.54	9.69 ± 1.29	9.22 ± 1.53	0.390; 0.002	≤ 0.001***; 0.146	0.019*; 0.020
Dimension Ex–N (dx)	1–5	49.18 ± 2.50	48.34 ± 2.79	48.12 ± 2.64	47.86 ± 2.14	52.55 ± 2.88	51.63 ± 2.68	50.92 ± 2.42	48.54 ± 2.14	≤ 0.001***; 0.249	≤ 0.001***; 0.079	≤ 0.001**; 0.031
Dimension Ex–N (sin)	4–5	48.59 ± 2.50	48.55 ± 2.79	48.04 ± 2.55	46.66 ± 2.29	51.94 ± 2.68	51.98 ± 2.75	50.60 ± 2.64	48.08 ± 1.79	≤ 0.001***; 0.259	≤ 0.001***; 0.118	0.011*; 0.018
Nasal length	5–6	42.72 ± 3.32	44.90 ± 3.21	45.92 ± 3.43	44.82 ± 3.88	45.51 ± 5.16	48.63 ± 3.72	49.22 ± 3.83	49.53 ± 2.81	≤ 0.001***; 0.164	≤ 0.001***; 0.109	0216; 0.006
Nasal width	10–11	29.63 ± 2.89	31.62 ± 2.59	33.03 ± 2.84	34.41 ± 2.62	32.91 ± 2.91	34.72 ± 2.83	37.59 ± 2.86	37.38 ± 2.57	≤ 0.001***; 0.249	≤ 0.001***; 0.281	0.014*; 0.014
Nasal depth	6–16	16.99 ± 2.27	18.84 ± 1.92	19.98 ± 1.99	20.38 ± 2.34	18.53 ± 2.21	19.99 ± 1.85	21.83 ± 2.29	23.16 ± 2.45	≤ 0.001***; 0.098	≤ 0.001***; 0.292	0.024*; 0.015
Philtrum hight	16–17	15.01 ± 1.86	15.14 ± 2.10	16.25 ± 2.09	16.61 ± 2.24	16.56 ± 2.21	16.76 ± 2.67	18.28 ± 2.35	19.63 ± 3.27	≤ 0.001***; 0.126	≤ 0.001***; 0.116	0.084; 0.011
Nasal height	5–16	48.21 ± 3.20	49.48 ± 3.58	50.16 ± 2.93	51.93 ± 3.28	51.40 ± 4.88	53.91 ± 3.80	54.43 ± 3.45	54.26 ± 2.56	≤ 0.001***; 0.192	≤ 0.001***; 0.086	0.270; 0.005
Dimension Pro-Po	6–9	67.17 ± 4.09	66.99 ± 4.82	66.28 ± 5.15	66.10 ± 4.66	72.30 ± 4.68	71.44 ± 5.48	72.39 ± 6.35	71.81 ± 5.67	≤ 0.001***; 0.213	0.133; 0.003	0.293; 0.004
Mouth width	7–8	47.19 ± 3.26	48.09 ± 3.35	48.83 ± 3.70	50.63 ± 3.82	49.81 ± 4.28	51.28 ± 3.81	53.79 ± 4.38	52.68 ± 4.31	≤ 0.001***; 0.145	≤ 0.001***; 0.094	0.015*; 0.020
Mouth height	17–18	15.69 ± 2.82	14.64 ± 2.40	12.28 ± 2.70	11.01 ± 2.24	16.07 ± 3.14	14.68 ± 3.15	11.88 ± 2.66	9.17 ± 1.94	0.543; 0.001	≤ 0.001***; 0.348	0.014*; 0.016
Facial height	5–9	99.36 ± 4.50	100.83 ± 5.02	101.24 ± 6.00	100.89 ± 5.34	105.77 ± 7.06	107.98 ± 6.14	109.67 ± 6.91	110.46 ± 5.74	≤ 0.001***; 0.286	0.030*; 0.034	0.090; 0.008
Lower face height	9–16	53.19 ± 3.51	52.19 ± 4.42	51.82 ± 3.89	52.10 ± 3.71	56.89 ± 4.90	55.89 ± 4.84	56.42 ± 5.60	56.75 ± 5.28	≤ 0.001***; 0.178	0.061; 0.009	0.322; 0.00
Facial width	19–20	109.59 ± 5.68	109.44 ± 6.45	112.65 ± 6.47	114.14 ± 6.46	119.98 ± 7.75	118.37 ± 5.14	120.66 ± 5.71	119.31 ± 5.10	≤ 0.001***; 0.299	0.007**; 0.045	0.015*; 0.016

In general, facial morphology was significantly affected by aging (with large or medium effect sizes, with exceptions such as biocular and facial width, Pro—Po dimension, and facial and lower facial height). The impact of age on facial variables was not confirmed only in lower facial heights (9–16, 6–9). Sexual dimorphism was apparent in almost all dimensions evaluated throughout the face, with large or medium effect sizes, except for some ocular dimensions, mouth height, ocular height (12–13, 14–15) and mouth width (17–18). Overall, the male faces were significantly larger in all the evaluated facial dimensions in comparison with the female faces. Finally, Tukey’s post hoc test helped interpret the results of the analysis of the interaction of sex and age on the facial variables, with the interactions having only a small effect size.

Eye slit widths (1–2, 3–4) narrowed significantly during aging, which also led to a significant diminishing of biocular width (1–4). Contrarily, intercanthal width (2–3) significantly widened during aging due to the reduction of ocular width and the increase in the distance between the eyes. Decreasing distances between Ex–N (1–5, 4–5) confirmed the narrowing of the eye slits during aging. Ocular height (12–13, 14–15) manifested a declining tendency in both sexes. The narrowing of both eye slit heights was not significant between early adulthood (I) and middle adulthood (II). In summary, in the ocular area, there was a gradual decrease of the ocular slit as a whole, while the interocular distance increased from adolescence to elderly age.

In the ocular region, sex-related differences were demonstrated in all the analyzed dimensions with the exception of ocular heights (12–13, 14–15). Overall, sexual dimorphism became insignificant in all ocular dimensions in elderly age (III). Interestingly, in the ocular widths (1–2, 3–4), the sexual dimorphism diminished significantly from middle adulthood (II) onward.

The nasal length (5–6) elongated during aging in both sexes, significantly only between juvenile age (0) and the other age categories (I, II, III). Nasal width (10–11) and nasal depth (6–16) increased significantly throughout the whole of adulthood, except for nasal width in the period between middle adulthood (II) and elderly age (III). The dimension between Pro–Po (6–9) decreased in women during the whole aging process, while it decreased in men only between the juvenile age (0) and early adulthood (I), and again between middle adulthood (II) and elderly age (III). This modeled growth tendency was not significant among any age category. Overall, the nose lengthened and widened during aging, while the nasal tip dropped downwards.

In the nasal region, all evaluated dimensions were larger in males compared to females. The sexual dimorphism appeared in all dimensions and remained apparent in the elderly age.

The mouth width (7–8) widened during the observed period; nevertheless, this modeled growth tendency was not apparent between juvenile age (0) and early adulthood (I), or between middle adulthood (II) and elderly age (III). Philtrum height (16–17) increased possibly due to the narrowing of the upper lip and/or decrease of the nasal tip; however, this modeled growth trend was also insignificant in the periods between juvenile age (0) and early adulthood (I) and between middle adulthood (II) and elderly age (III). In contrast, mouth height (17–18) significantly decreased with aging among all the analyzed age categories. It followed that the mouth lengthened and narrowed during the modeled development.

In the orolabial region, sexual dimorphism was not manifested in mouth height (17–18). Intersex differences were demonstrated in the mouth width (7–8) and philtrum height (16–17); however, the sexual dimorphism gradually disappeared in elderly age (III).

As for the overall dimensions of the face, facial height (5–9) enlarged during adulthood, significantly between juvenile age (0) and middle adulthood (II). The modeled growth changes in lower facial height (9–16) were ambiguous in men as well as in women. In both sexes, the lower facial height shortened at first and then lengthened again; in women, the dimension shortened up to middle adulthood (II), while in the elderly age (III) it lengthened again. In men, the lower facial height increased from middle adulthood (II). Nonetheless, this modeled growth trend in lower facial height was not significant between any age categories. Facial width (19–20) widened significantly between early (I) and middle adulthood (II), more clearly in women. The modeled trend was not unambiguous in men; however, the face widened during aging as it did in women.

## Discussion

The human face is a complex and dynamic system affected by aging, sex, health condition, BMI, expressed emotions and many other features^36,37^. It is well known that according to sex, the adult human face varies significantly in both hard and soft facial tissue^38^. These sex-related facial differences develop throughout the whole of adulthood up to elderly age^39^. In this study, the soft tissue surface as a whole and dimensions have been found to modify between adolescence, youth, middle adulthood, and elderly age. The presented data were cross-sectional, therefore, do not represent real growth or aging, but only modeled estimates, because different groups of subjects were examined at different ages. The possible presence of secular trends should be considered^40^.

Concerning the evaluation of modeled facial development as a whole, aging in both sexes shared common traits, such as more pronounced facial roundness (more rectangular in males), decreased facial convexity, narrower eye slits and thinner lips, increased visibility of skin folds and wrinkles connected with the loss of skin elasticity, and soft tissue stretching, especially in the orbital area and lower face (“broken” jawline).

According to our results, between juvenile age and younger adulthood, a woman’s face does not change, while in men it lengthens, with especially the chin area being emphasized. In elderly adulthood its width increases, more significantly in the lower part of the cheeks. Our findings resemble the 3D visualizations of facial aging from Chinese and Croatian samples^41,42^.

Aging was generally associated with a flatter face^13,41^, which means a reduction in the prominence of the superciliary arch, nose, and lip area, although some facial dimensions increase with age (e.g. width and height of face, nose, and distance between nose and mouth, which will be discussed in the following text). Furthermore, the midface is described as an area with great bone resorpsion, where the orbital aperture and apertura piriformis increase with age^9,39^. This aging manifested in the surface changes of the soft tissues of the face in this study is also consistent with the age-related changes in the splanchnocranium CT images of the skulls^33^ of the current Czech population.

Facial morphology was affected by sex and age in almost all the variables evaluated in this study. It is very well documented^32,38,43^ that the face generally manifested strong sexual dimorphism in height and width dimensions, which was also confirmed by the results of our study. Generally, the male faces were bigger and wider than the female faces. In the presented sample, sexual dimorphism was apparent in almost all the facial parameters, with more pronounced and large features in men, which is supported by many previous studies^38,43,44^. When the whole face is taken into account, the vertical and horizontal facial dimensions were larger and more pronounced in men, which corresponds to the conclusions of Liu et al. (2014)^43^, even though they compared different ethnicities from those of our sample.

Almost all the ocular dimensions diminished during aging, with the exception of the intercanthal width, which was confirmed by the increased distance between the eyes. In our case, a clear downward trend was observed in ocular height, which was contrary to several authors^45,46^ who discovered a slight increase in this dimension. This observed trend could be explained by the overall reduction of the eye with advancing age^47^, or by the lowering position of the eyelid^12^.

Linear dimensions in the ocular region, except for ocular height, showed sex-related differences. Some other studies reached the same outcome, even though the dimensions in the ocular region were analyzed in different ways compared to those in this article^45,48^. According to the previous statement, ocular height was a sex independent dimension affected primarily by aging. In opposition to our outcomes, Modabber et al. (2020)^46^ found significantly higher eye slits in women, in line with Farkas et al. (2005)^49^. In Liu et al. (2014)^43^, the sex-related differences were not observed in intercanthal width, which was not consistent with our conclusions. However, it is highly probable that this discrepancy was caused by the study being done with different ethnicities. In contrast to previous findings, no evidence of intersex differences in the ocular area were manifested in Gupta et al. (2003)^50^, although a different ethnic group or age distribution may cause this dissention.

When it comes to the modeled growth changes in the nasolabial area, older people were inclined to manifest larger, longer and wider noses compared to younger individuals^46,51^. While the nose lengthened and grew even during the elderly period, according to Modabber et al. (2020)^46^, the thickness of the nasal wings remained constant throughout life. This was inconsistent with our results, where the width of the nasal wings broadened during aging. Significant increases in almost all nasal dimensions were observed in our case in early and middle adulthood up to 40 years of age; however, the increasing tendency slowed during the elderly age. Although nasal cartilage grows throughout life^52^, Sforza et al. (2010)^53^ reported findings that were in line with our outcomes: that the most significant growth changes in the nasal area appeared in childhood, adolescence and early adulthood. The growth of the nasal area continued even after the age of 20; however, at a very slow rate, as confirmed by a number of previous studies^51,54^. In the nasal region, soft tissue is slightly modified in older age regardless of ethnicity, in accordance with muscle or cartilage changes, skin elasticity, and many others factors explaining the less pronounced modeled growth tendencies^12,53^. In contrast, the investigation of Torlakovic and Faerovig (2011)^55^ did not note any significant changes in the nasal area after the age of 20.

The nasal region tended to be sexually dimorphic and significantly affected by increasing age, similar to the outcomes summarized in Sforza et al. (2010)^53^. Men had larger nasal dimensions than women, which was confirmed by many other studies involving a range of various ethnic groups^46,51,52^. According to several authors, the soft-tissue growth of the nose or the physiologically higher oxygen requirements in men were the most crucial factors responsible for the intersex differences^56,57^.

The orolabial area was also significantly influenced by aging, manifested mainly by the narrowing of the mouth, reduction of lip thickness, and increasing distance between nose and mouth (philtrum height)^12,58^. These elderly age modifications were also observed in the group of individuals in our study. In the analyzed groups, narrowing of the lips was detected, similarly to the longitudinal study by Akgül and Toygar (2002)^59^. Lip modifications during senescence may be caused by post-menopausal hormonal changes in women, lip tonicity, reduced elastic fibrils, reduction in the vermilion border, disappearance of Cupid’s bow, or loss of elasticity and skin thickness^58^. During old age, there was progressive elongation of the lips, in agreement with several publications^47,60^, which could affect oral or dental aesthetics. There was a significant increase even in middle adulthood, unlike Sforza et al. (2010)^5^, who observed an increase primarily at a younger age.

Similar to the other parts of the face, the orolabial area was sexually dimorphic with larger and more pronounced mouth features in men, as reported in much previous literature^5,56,61^. In general, these results may be caused due to a different body and muscle composition according to sex, age, or ethnicity. However, mouth height was not sexually dimorphic in our set of individuals. The size of the dimension was comparable between men and women, although some studies suggested that women’s lips were narrower, especially in childhood and adolescence^61^.

The lower facial height appeared to lengthen during aging, for example, due to a descent of the interlabial line and a reduction of the upper lip. Sharma et al. (2014)^62^ stated that lower facial height increased in men, while remaining roughly the same in women, due to a slight increase in the maxillary/mandibular plane angle. This was only partially in consensus with our results, whereby an increase in lower facial height was found in men from middle adulthood. In conclusion, older people had wider and longer faces according to our findings, which is supported by several studies^32,44^.

## Conclusion

This paper presents the modeling of age-related facial changes from juvenile to elderly age based on transversal data (a Central European set of 456 3D surface models of the human face).

In association with increasing age, there is prominence of the forehead, the nose and lip area decrease, while the face widens laterally, especially in the buccal area. The modeled facial development have a similar course between 30 and 70 years of age in both women and men, although we recorded some differences and more pronounced male changes during the whole investigated period. Only male’s faces increase until almost 30 years of age. After the age of 70, male facial size not only stagnates, like in females, but it decreases slightly.

When monitoring the average differences between age categories, there were no significant morphological changes in females from juvenile age to younger adulthood. In men, on the other hand, some signs of sexual dimorphism, such as prominence of the eyebrows, nose, and chin, became more pronounced during this period. In the following period, we did not notice any significant differences in facial aging between the sexes and their facial aging pattern did not diverge after menopause. After the age of 60, the sagging soft tissue in the lateral region of the manibula was highlighted. When it comes to the metric evaluation of the face, most dimensions increased with age in the observed period; however, the eye slit width and height narrowed significantly during aging. Similarly, mouth height significantly decreased with aging in all the analyzed age categories.

Significant sexual dimorphism was found in almost all dimensions evaluated throughout the face, except for ocular and mouth height. Sexual dimorphism decreased in the frontal and orbitonasal regions, while in the lateral region of the mandible, it increased with age.

## Supplementary Information

Supplementary Table 1.

## Data Availability

The datasets generated and analysed during the current study are not publicly available due to informed consent being signed specifically for the purpose of the study; however, they are available from the corresponding author on reasonable request.
