# Supplementary material for: Three-dimensional analysis of modeled facial aging and sexual dimorphism from juvenile to elderly age
Source: Sci Rep. 2022 Dec 17;12:21821. doi: 10.1038/s41598-022-26376-8 (PMC9759541; doi:10.1038/s41598-022-26376-8)
Supplement: Supplementary file 1 — Supplementary Table 1. [file 41598_2022_26376_MOESM1_ESM.docx]

**Supplementary table S1**: Result of Tukey’s post hoc test expressing modelled growth trends between age categories. NS = not statistically significant.

| Dimensions | Dim. | T0xT1 | T0xT2 | T0xT3 | T1xT2 | T1xT3 | T2xT3 |
| --- | --- | --- | --- | --- | --- | --- | --- |
| Ocular width (dx) (dx) | 1–2 | 0.033 | 0 | 0 | ≤0.001 | 0 | 0 |
| Ocular width (sin) | 3–4 | NS | ≤0.001 | 0 | ≤0.001 | 0 | 0 |
| Intercanthal width | 2–3 | NS | 0.011 | ≤0.001 | ≤0.001 | 0 | ≤0.001 |
| Biocular width | 1–4 | NS | 0.010 | ≤0.001 | NS | ≤0.001 | ≤0.001 |
| Ocular height (dx) | 12–13 | ≤0.001 | ≤0.001 | ≤0.001 | NS | 0.006 | NS |
| Ocular height (sin) | 14–15 | ≤0.001 | ≤0.001 | 0 | NS | ≤0.001 | 0.004 |
| Dimension Ex–N (dx) | 1–5 | 0.013 | ≤0.001 | 0 | NS | ≤0.001 | 0.001 |
| Dimension Ex–N (sin) | 4–5 | NS | 0.003 | 0 | 0.008 | 0 | ≤0.001 |
| Nasal length | 5–6 | ≤0.001 | ≤0.001 | ≤0.001 | NS | NS | NS |
| Nasal width | 10–11 | ≤0.001 | 0 | 0 | ≤0.001 | ≤0.001 | NS |
| Nasal depth | 6–16 | ≤0.001 | 0 | 0 | ≤0.001 | ≤0.001 | NS |
| Philtrum hight | 16–17 | NS | ≤0.001 | ≤0.001 | ≤0.001 | ≤0.001 | NS |
| Nasal height | 5–16 | ≤0.001 | ≤0.001 | NS | NS | ≤0.001 | 0.017 |
| Dimension Pro-Po | 6–9 | NS | NS | NS | NS | NS | NS |
| Mouth width | 7–8 | NS | ≤0.001 | ≤0.001 | 0.013 | 0.011 | NS |
| Mouth height | 17–18 | 0.004 | 0 | 0 | 0 | 0 | ≤0.001 |
| Facial height | 5–9 | NS | 0.015 | NS | NS | NS | NS |
| Lower face height | 9–16 | NS | NS | NS | NS | NS | NS |
| Facial width | 19–20 | NS | NS | NS | 0.005 | NS | NS |
